# Involvement of Potassium Channels in Vasorelaxant Effect Induced by *Valeriana prionophylla* Standl. in Rat Mesenteric Artery

**DOI:** 10.1155/2013/147670

**Published:** 2013-08-14

**Authors:** Milena Ramos Reis, Abrahão Alves de Oliveira Filho, Lilia Simone Urzedo Rodrigues, Jaíse Paiva Araújo, Priscilla Maria Pereira Maciel, Jamile Morais de Albuquerque, Valdir Cehinel Filho, Armando Cáceres, Josmara Bartolomei Fregoneze, Isac Almeida de Medeiros, Darizy Flávia Silva

**Affiliations:** ^1^Laboratório de Fisiologia e Farmacologia Endócrina e Cardiovascular, Departamento de Biorregulação, Instituto de Ciências da Saúde, Universidade Federal da Bahia, Avenida Reitor Miguel Calmon, Vale do Canela, 40110-902 Salvador, BA, Brazil; ^2^Laboratório de Farmacologia Cardiovascular, Centro de Biotecnologia, Universidade Federal da Paraíba, Cidade Universitária, 58051-900 João Pessoa, PB, Brazil; ^3^Laboratório de Neurociências, Instituto de Ciências da Saúde, Universidade Federal da Bahia, Avenida Reitor Miguel Calmon, Vale do Canela, 40110-902 Salvador, BA, Brazil; ^4^Núcleo de Investigações Químico-Farmacêuticas, Centro de Ciências da Saúde, Universidade do Vale do Itajaí, Rua Uruguai, 458 Centro, 88302-202 Itajaí, SC, Brazil; ^5^Facultad de Ciencias Químicas y Farmacia, Universidad de San Carlos de Guatemala (USAC), 01012 Ciudad de Guatemala, Guatemala

## Abstract

Assays *in vitro* and *in vivo* were performed on extract from roots and leaves from the *Valeriana prionophylla *Standl. (VPR and VPF, resp.). In phenylephrine (1 **μ**M) precontracted rings, VPR (0.01–300 **μ**g/mL) induced a concentration-dependent relaxation (maximum response (MR) = 75.4 ± 4.0%, EC_50_ = 5.97 (3.8–9.3) **μ**g/mL, *n* = 6]); this effect was significantly modified after removal of the endothelium (EC_50_ = 39.6 (27.2–57.6) **μ**g/mL, *P* < 0.05). However, VPF-induced vasorelaxation was less effective compared to VPR. When rings were preincubated with L-NAME (100 **μ**M) or indomethacin (10 **μ**M), the endothelium-dependent relaxation induced by VPR was significantly attenuated (MR = 20.9 ± 2.3%, 34.2 ± 2.9%, resp., *P* < 0.001). In rings denuded endothelium, precontracted with KCl (80 mM), or in preparations pretreated with KCl (20 mM) or tetraethylammonium (1 or 3 mM), the vasorelaxant activity of VPR was significantly attenuated (MR = 40.0 ± 8.2, *n* = 5; 50.5 ± 6.0%; 49.3 ± 6.4%; 46.8 ± 6.2%; resp., *P* < 0.01). In contrast, neither glibenclamide (10 **μ**M), barium chloride (30 **μ**M), nor 4-aminopyridine (1 mM) affected VPR-induced relaxation. Taken together, these results demonstrate that hypotension induced by VPR seems to involve, at least in part, a vascular component. Furthermore, endothelium-independent relaxation induced by VPR involves K^+^ channels activation, most likely due to BK_Ca_ channels, in the rat superior mesenteric artery.

## 1. Introduction

The Valerianaceae family is well known for an abundance of different species of the genus *Valeriana *L. used in the folk medicine for the treatment of psychosomatic disorders, such as anxiety and insomnia [1, 2]. *Valeriana prionophylla* Standl. is a species distributed throughout Latin American, mainly Costa Rica, Guatemala, and Mexico, and is known as “Valeriana del monte.” Studies performed in animals have demonstrated that rhizomes of this species affect central nervous system activity, demonstrating sedative, hypnotic, anxiolytic, and antidepressive effects [3, 4]. However, despite its widespread use by the population for psychosomatic disorders, possible peripheral effect of this species is not well characterized; it is important to investigate a possible peripheral cardiovascular action and thus differentiate between central and peripheral effects in the cardiovascular system.

Furthermore, phytochemical analysis of rhizomes of *V. prionophylla* identified the presence of valepotriates [5], and new lignans isolated from these roots have demonstrated vasorelaxant activity in isolated rat aorta artery rings [6] demonstrating a potential effect on peripheral systems. Lignans are secondary plant metabolites that exist in the phenylpropanoid pathway and have been identified and isolated from approximately 70 different families of all plant origins, the majority of which are those used in popular medicine [7]. Lignans in the cardiovascular system have demonstrated a therapeutic potential as a cardiotonic agent, by the inhibition of phosphodiesterase III, which is responsible for metabolizing the second messenger cAMP (3.5-cyclic adenosine monophosphate). Moreover, some lignans also demonstrate antagonistic activity of the platelet-activating factor (PAF) receptor and blocking of the L-type Ca^2+^ channel [8].

Many cardiovascular disorders, such as hypertension, angina, and heart failure, are often treated with vasodilator drugs that act directly on the vascular smooth muscle, causing vasodilation, indirectly by stimulating the release of endogenous vasorelaxant factors or by inhibiting the release of vasoconstrictive factors [9].

Thus, the aim of this study was to evaluate the peripheral actions of *Valeriana prionophylla* Standl. in the cardiovascular system and the mechanisms underlying the vascular response induced by this species in isolated rat mesenteric artery.

## 2. Materials and Methods

### 2.1. Drugs and Solutions

The drugs used in this study were Cremophor EL, dimethyl sulphoxide (DMSO), L-phenylephrine chloride (Phe), acetylcholine chloride (Ach), glibenclamide, tetraethylammonium, 4-aminopyridine, and barium chloride (SIGMA). All compounds were dissolved in distilled water, except glibenclamide that was dissolved in DMSO. The composition of Tyrode's solution used was as follows (mM): NaCl, 158.3; KCl, 4.0; CaCl_2_, 2.0; MgCl_2_, 1.05; NaH_2_PO_4_, 0.42; NaHCO_3_, 10.0, and glucose, 5.6. K^+^-depolarizing solutions (KCl 20 and 80 mM) were prepared by replacing 20 or 80 mM KCl in Tyrode's solution with equimolar NaCl, respectively.

### 2.2. Plant Material

*Valeriana prionophylla* Standl., Valerianaceae, were collected from cultivations in Tierra Blanca, Concepción Tutuapa, San Marcos (15° 14.808′N, 91° 55.430′W), Guatemala, a vegetative zone that resides in a very humid and low mountainous forest. Three-year-old rhizomes and roots were dug up, washed, and shade-dried. Botanical samples were determined by Mario Veliz at Herbarium BIGU, School of Biology, USAC, and a voucher sample deposited (no. 49183). 

### 2.3. Preparation of Ethanol Extract

Dry material was ground, wetted with 50% ethanol, and placed in a stainless steel percolator. 50% ethanol was added to obtain a tincture, which was concentrated in a rotavapor. Fresh ethanol was added for five consecutive days, and the extract was concentrated. The final drying was performed in a vacuum dryer with silica gel, as described by Holzmann et al. [3]. The average yield of the extractable solids was 28.52%. For *in vitro* experiments, extracts of the roots and leaves from the *Valeriana prionophylla* Standl. species (VPR and VPF, resp.) were dissolved in a mixture of distilled water/Cremophor and diluted to the desired concentrations with distilled water just before use; the final concentration of Cremophor in the bath never exceeded 0.01%.

### 2.4. Animals

Male Wistar rats (250–300 g) were used for all experiments. Animals were housed under controlled temperature (21 ± 1°C), exposed to a 12 h light-dark cycle with free access to food (Purina, Brazil) and tap water. The study was carried out in accordance with the Guide for the Care and Use of Laboratory Animals as adopted by the US National Institutes of Health.

### 2.5. Tissue Preparation

Rats were euthanized, and superior mesenteric artery was removed, cleaned from connective tissue and fat as described by Silva and colleagues [10]. Whenever appropriate, the endothelium was removed by gently rubbing the intimal surface of the vessels. Rings (1-2 mm) were suspended in organ baths containing 10 mL of Tyrode's solution, gassed with a mixture of 95% O_2_ and 5% CO_2_, maintained at 37°C and at pH 7.4. Isometric tension was recorded under a resting tension of 0.75 g. The solution was changed every 15 min during a stabilization period of 1 hr to prevent the accumulation of metabolites [11]. The isometric contractile force was recorded by a force transducer (MLT020, ADInstruments, Australia) coupled to an amplifier-recorder (ML870/P com LabChart versão 7.0, ADInstruments, Australia) and to a computer equipped with a data acquisition software. The presence of functional endothelium was assessed by the ability of Ach (10 *μ*M) to induce more than 90% relaxation of pre-contracted vessels with Phe (10 *μ*M) and the absence, less than 10% of relaxation induced by Ach.

### 2.6. Effects of VPF and VPR in Phenylephrine-Induced Contractions

In this experiment, sustained Phe-induced contractions were obtained in isolated rat superior mesenteric artery rings with or without endothelium. In the tonic phase of the second contraction induced by Phe (1 *μ*M), increasing cumulative concentrations of VPF and VPR (0.01; 0.03; 0.1; 0.3; 1; 3; 10; 30; 100; and 300 *μ*g/mL) were separately and continually added to the bath until a maximum response for the added extract was observed, as indicated by a plateau response (approximately 4–6 min). 

### 2.7. Investigation of Nitric Oxide and COX-Derived Products Participation in the Vasorelaxation Mediated by VPR

To investigate the involvement of NO and prostanoids, endothelium-intact rings were incubated for 30 min with L-NAME (10^−4^ M) or indomethacin (10^−5^ M) (endothelial nitric oxide synthase (eNOS) or cyclooxygenase inhibitors, resp.) before the second application of Phe (1 *μ*M), and the vasorelaxation induced by VPR (300 *μ*g/mL) was investigated before and after the addition of the inhibitors.

### 2.8. Effect of VPR on Contraction Induced by Depolarization with High K^+^ Concentration

After the stabilization period, rings without endothelium were precontracted with high potassium concentration solution (KCl 80 mM), and, in the tonic phase, different concentrations of VPR (0.01–300 *μ*g/mL) were added cumulatively to the organ bath, and relaxations were measured, as previously described. 

### 2.9. Investigation of K^+^ Channel Participation in the Vasorelaxation Elicited by VPR

To investigate the involvement of K^+^ channels, in the first set of experiments, vasorelaxation with VPR was performed in vessels with denuded endothelium that were precontracted with Phe in Tyrode's solution with elevated K^+^ (20 mM equimolar replacement of NaCl with KCl) to attenuate K^+^ efflux [10]. Additionally, in the second group of experiments, endothelium-denuded rings were incubated for 30 min with putative K^+^ channel blockers before the second application of Phe (1 *μ*M), and the inhibition was calculated by comparing the response elicited by VPR, before and after the addition of the inhibitors. Preparations were exposed to tetraethylammonium (TEA, 3 mM), a nonselective K^+^ channel blocker; TEA (1 mM), a large-conductance calcium-activated K^+^ channel (BK_Ca_) selective blocker in this concentration; 4-aminopyridine (4-AP) (1 mM), a voltage-dependent K^+^ (K_*v*_) channel blocker; glibenclamide (Glib) (10 *μ*M), an ATP-sensitive K^+^ (K_ATP_) channel blocker; and barium chloride (BaCl_2_) (30 *μ*M), an inward rectifier K^+^ (K_ir_) channel blocker. 

### 2.10. Investigation of Effect of VPR on CaCl_2_-Induced Contractions

To investigate the hypothesis that VPR act through the blockade of extracellular calcium influx in endothelium-denuded rings, cumulative concentrations of CaCl_2_ (10^−6^–10^−2^ M) were added in medium containing Ca^2+^-free depolarizing solution (KCl, 60 mM) in absence (control) or in presence of VPR (300 *μ*g/mL). 

### 2.11. Measurement of Mean Arterial Pressure and Heart Rate in Nonanesthetized Normotensive Rats

The day before the experimental session, a catheter (PE_50_) filled with heparinized saline solution (1000 U/mL) was inserted into the left carotid artery under ketamine/xylazine anesthesia and exteriorized at the nape of the animal's neck to permit blood pressure recording. An additional catheter was placed in the right femoral vein to allow intravenous drug administration. After 24 hours, arterial pressure was continuously monitored through the carotid catheter connected to a blood pressure transducer (World Precision Instruments) whose signal was amplified and digitally recorded by an analog-to-digital interface (AqDados, application for data acquisition, Lynx Tecnologia Eletrônica Ltda, version 7.0, São Paulo, Brazil) and recorded (1 kHz) on a microcomputer for later analysis. Mean arterial pressure (MAP) was calculated from systolic and diastolic pressure data, while heart rate (HR) was determined by the pulsation of arterial pressure using the AcqKnowledge software program, version 3.5.7, developed by Biopac Systems, Inc., California, USA.

### 2.12. Statistical Analysis

Data are presented as the mean ± SEM, and *n* represents a number of rings prepared from different rats. Concentration-response curves to VPR and VPF were based on the percent relaxation of the agonist-induced contraction. A 100% relaxation was assigned when the precontracted rings returned to the baseline values. The curves were fitted using a variable slope sigmoid fitting routine in GraphPad Prism5.0 (Graph Pad Software, Inc., La Jolla, CA, USA). EC_50_ (concentration required to relax the induced tone by half, and maximum response (MR) values were calculated from the fitted sigmoidal curves. Statistical analyses were performed by comparing *E*_max⁡_ and EC_50_ values between groups from independent observations. Unpaired Student's *t*-test or one-way ANOVAs were used to compare 2, 3, or more groups, respectively, with the addition of Bonferroni's multiple comparisons post-est. Two-sided *P* < 0.05 was considered statistically significant.

## 3. Results

### 3.1. Effects of VPR and VPF in Phenylephrine-Induced Contractions

**Figure 1:**
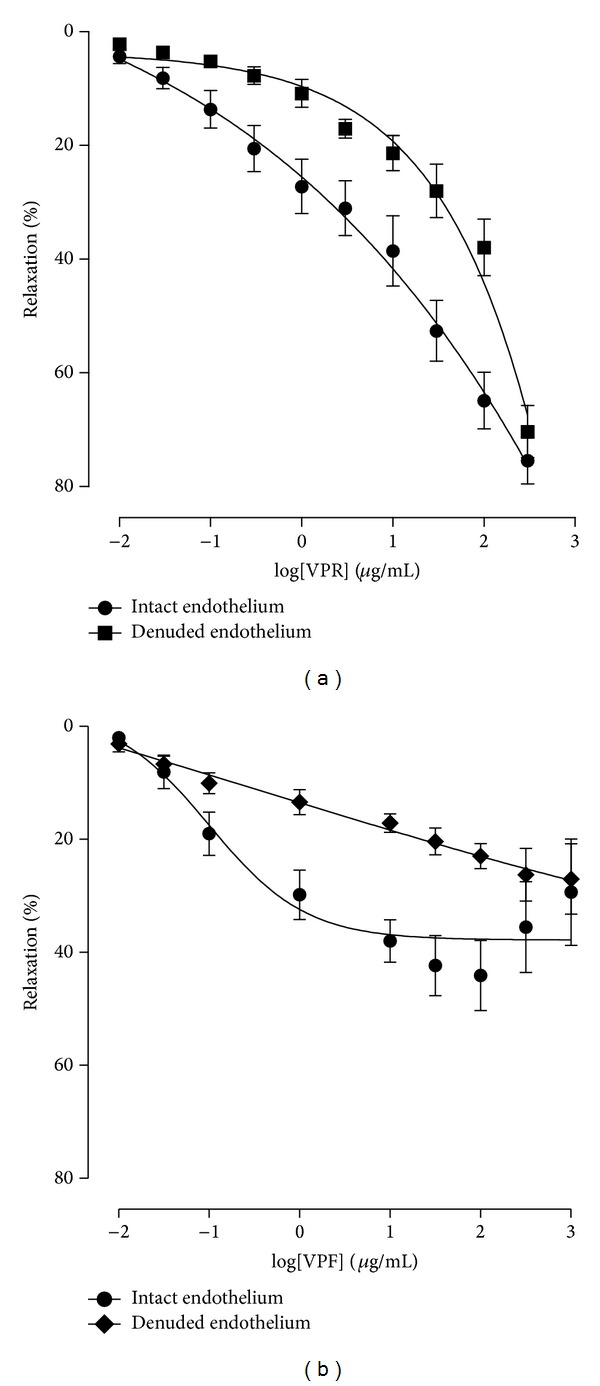
Vasorelaxant effect induced by VPR. Concentration-response curves to VPR or VPF (0.01–300 *μ*g/mL, cumulatively) precontracted with 1 *μ*M phenylephrine (Phe) in mesenteric artery rings (with endothelium) and after removal of endothelium: (a) concentration-response curves showing the relaxant effect of VPR, *n* = 6; (b) responses induced by VPF, *n* = 4. The response is expressed as a percentage relaxation of the Phe-induced contraction. Values are mean ± SEM.

In isolated rat superior mesenteric artery rings, VPR (0.01–300 *μ*g/mL) decreased in a concentration-dependent manner following Phe-induced contraction (1 *μ*M) (EC_50_ values = 5.96 (3.8−9.2) *μ*g/mL) (Figure 1(a)). In the absence of the vascular endothelium, the potency of VPR was significantly shifted (EC_50_ values = 39.63 (27.2−57.6) *μ*g/mL; *P* < 0.05) (Figure 1(a), Table 1), whereas VPF (0.01–300 *μ*g/mL) induced a vasorelaxant effect in the presence and absence of vascular endothelium (maximum response (MR) = 29.39 ± 9.4%, MR = 27.05 ± 6.2%, resp.; *n* = 4) (Figure 1(b)); however, this response was reduced in comparison to that obtained by VPR (MR = 75.43 ± 4.08%, MR = 70.33 ± 4.58%, *P* < 0.05, resp.).

### 3.2. Effect of VPR on Endothelium-Intact Rings in the Presence of L-NAME or Indomethacin

**Figure 2:**
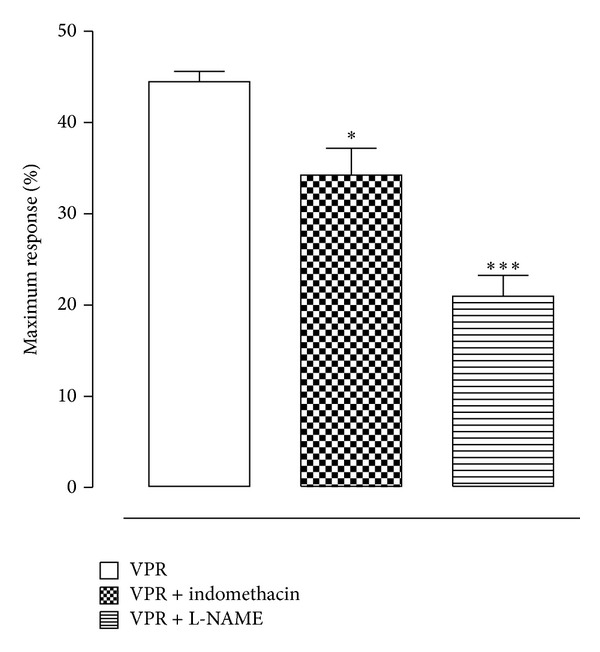
Participation of NO and COX-derived products in the vasorelaxation induced by VPR in mesenteric artery rings. Effect of VPR (300 *μ*g/mL) in rings intact endothelium precontracted with Phe (1 *μ*M) in the absence or in the presence of L-NAME (100 *μ*M) or indomethacin (10 *μ*M). Values are mean ± SEM; *n* = 5 and *n* = 5, respectively.

To investigate the involvement of NO and prostanoids, endothelium-intact rings were preincubated for 30 min with L-NAME (100 *μ*M) or indomethacin (10 *μ*M) before the second contraction with Phe (1 *μ*M).  Figure 2 shows that the vasorelaxant activity of VPR (300 *μ*g/mL) was significantly reduced in the presence of these inhibitors.

### 3.3. Effects of VPR on Contraction Induced by High K^+^ Concentration

**Figure 3:**
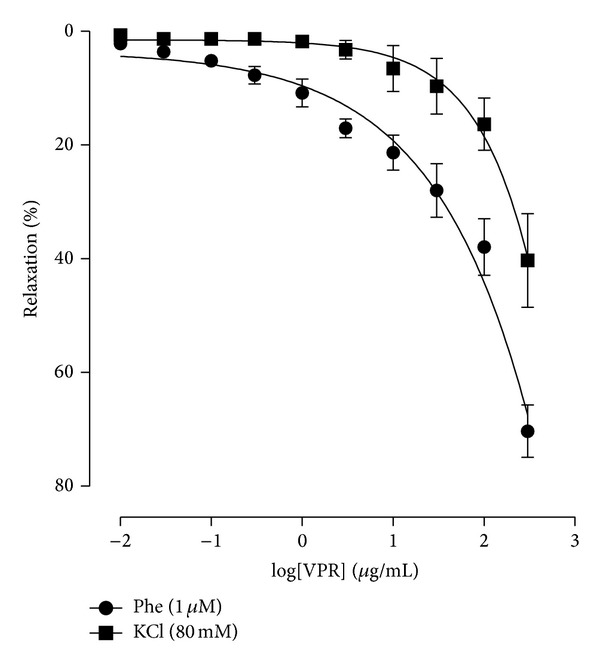
High K^+^ extracellular decreased relaxant effect of VPR. Concentration-response curves showing the relaxant effect of VPR (0.01–300 *μ*g/mL, cumulatively) in rings denuded endothelium contracted with Phe or KCl 80 mM. Values are expressed as mean ± SEM, *n* = 5.

As illustrated in Figure 3, following contraction with KCl 80 mM, VPR-mediated relaxation of endothelium-removed rings was significantly reduced when compared to the contractile response induced by Phe (Table 1), as demonstrated by the displacement of the concentration-response curve to the right.

### 3.4. Effect of VPR on Arteries Treated with KCl 20 mM

**Figure 4:**
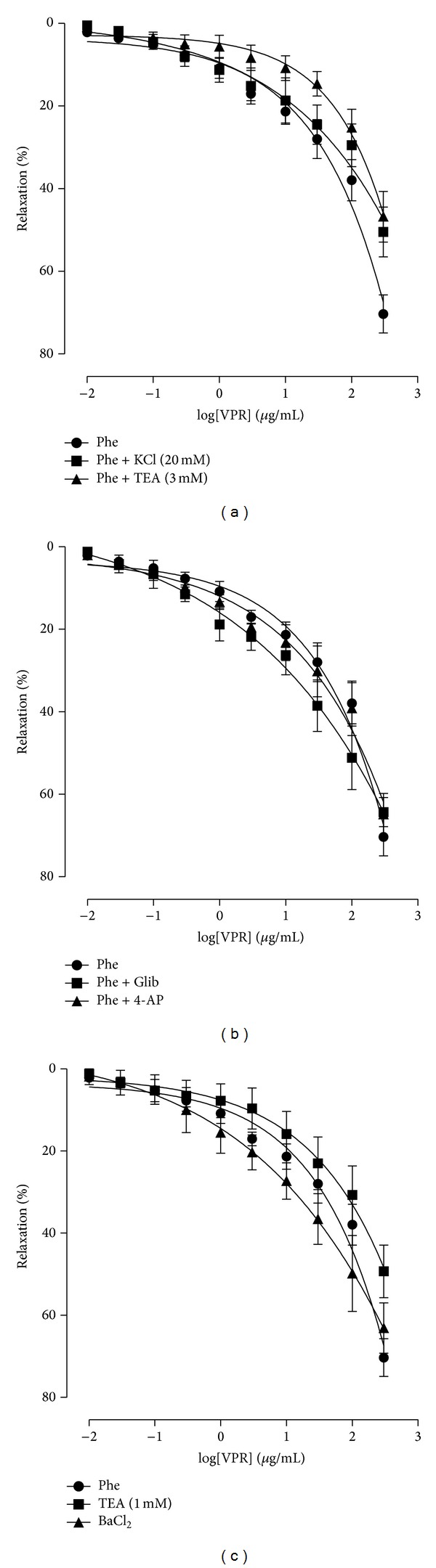
Participation of K^+^ channels in the vasorelaxation induced by VPR in mesenteric artery rings. Concentration-response curves showing the relaxant effect of VPR (0.01–300 *μ*g/mL, cumulatively) in rings denuded endothelium precontracted with Phe (1 *μ*M) in the absence or in the presence of (a) KCl 20 mM or TEA (3 mM); (b) 4-AP (1 mM) or Glib (10 *μ*M); (c) TEA (1 mM) or BaCl_2_ (30 *μ*M). Values are expressed as mean ± SEM.

To evaluate the involvement of K^+^ channels in the VPR-mediated vasorelaxant response, experiments were performed in endothelium-denuded rings incubated with KCl 20 mM and precontracted with Phe. As shown in Figure 4(a), the VPR-mediated vasorelaxation was reduced, with significant alterations in pharmacological efficacy when compared to the control (Table 1).

### 3.5. Effect of VPR on Endothelium-Denuded Rings in the Presence of Different K^+^ Channel Inhibitors

Figure 4(a) shows that the vasorelaxant activity of VPR at the highest concentration was significantly rightward shifted in the presence of TEA (3 mM) (Table 1). However, in the presence of Glib (10 *μ*M), 4-AP (1 mM), or BaCl_2_ (30 *μ*M), VPR-mediated relaxation in rings lacking the vascular endothelium and precontracted with Phe was not significantly reduced. In the presence of TEA (1 mM), the vasorelaxant response was significantly attenuated (Figures 4(b) and 4(c), Table 1).

### 3.6. Effect of VPR on CaCl_2_-Induced Contractions

**Figure 5:**
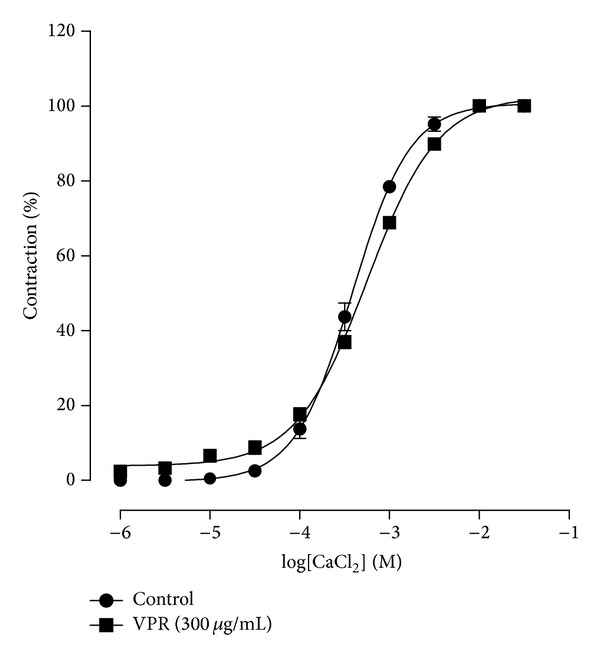
Effect of VPR on CaCl_2_-induced contraction in endothelium-denuded mesenteric artery rings. Concentration-response curves for CaCl_2_ were determined in Ca^2+^-free solution containing KCl (60 mM). The curves were determined in the absence (control) and after incubation with VPR (300 *μ*g/mL). Values are expressed as mean ± SEM, *n* = 4.

To investigate the hypothesis of the residual vasorelaxant effect induced by VPR being the same as in the presence of the K^+^ channels inhibitors, concentration-response curve was induced with CaCl_2_ in Ca^2+^-free depolarizing solution.  Figure 5 illustrates that VPR (300 *μ*g/mL) was not able to inhibit CaCl_2_-induced contractions, demonstrating that VPR extract probably does not inhibit extracellular calcium influx.

### 3.7. VPR Effect on MAP and HR in Nonanesthetized Rats

**Figure 6:**
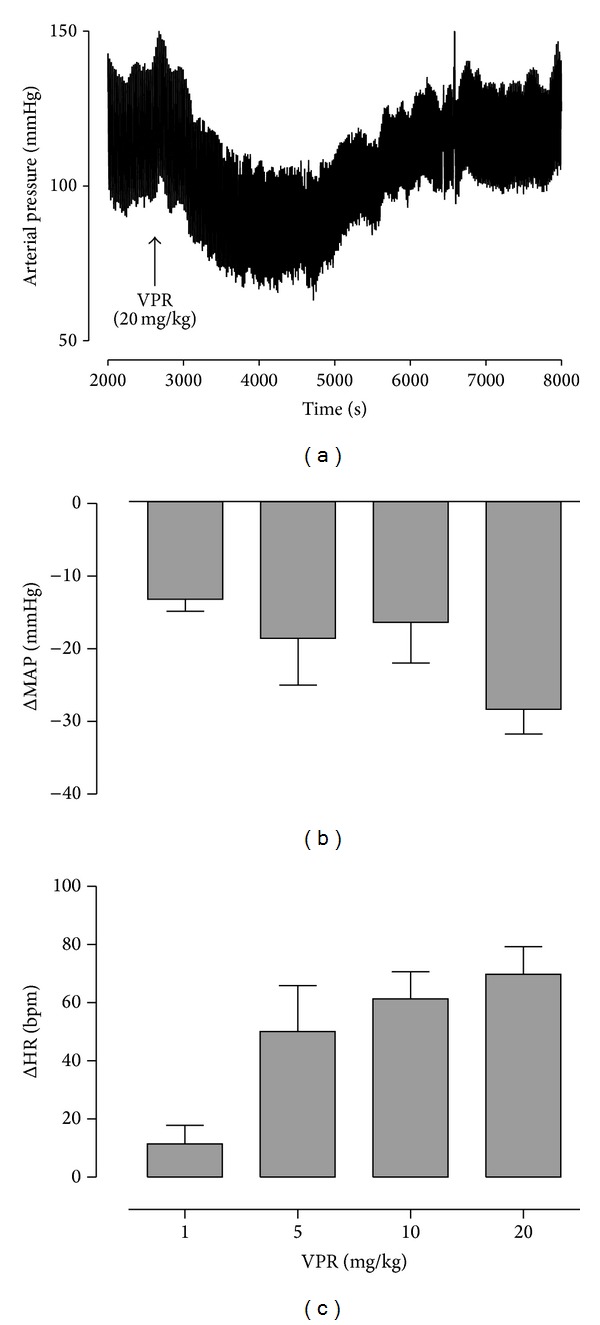
Effect of VPR on MAP and HR in nonanesthetized rats. (a) Representative tracing showed the hypotension induced by addition of VPR (20 mg/kg). (b) Changes in mean arterial pressure (MAP, mmHg). (c) Changes in heart rate (HR, bpm) induced by the acute administration of increasing doses of VPR (mg/kg, i.v). Values are expressed by mean ± SEM; *n* = 5.

As Figure 6 shows, in nonanesthetized normotensive rats, intravenous bolus injections of VPR (1, 5, 10, and 20 mg/kg) induced hypotension (MAP = −13.18 ± 1.6, −18.54 ± 6.4, −16.36 ± 5.6, and −28.32 ± 3.4 mmHg, resp.; *n* = 5) (Figures 6(a) and 6(b)) associated with tachycardia (HR = 11.64 ± 5.5; 50.24 ± 14.0; 61.45 ± 8.0; and 69.88 ± 8.4 bpm, resp.; *n* = 5) (Figure 6(c)).

## 4. Discussion

The biological effects of *Valeriana prionophylla* Standl. in the cardiovascular system have yet to be fully elucidated. This study aimed to increase our understanding of this natural medicine and highlight its potential pharmacological contribution. Specifically, this work demonstrated cardiovascular pharmacological effects of VPR in rats using *in vitro* and *in vivo* protocols.

Initially, we evaluated the peripheral vascular effects of VPR and VPF by performing *in vitro* experiments using rat superior mesenteric artery rings. VPR significantly reduce Phe-induced contractions, a selective *α*_1_-adrenergic receptor agonist, in a concentration-dependent manner [12]. VPF also induced a vasorelaxant effect in mesenteric artery rings, but this response was reduced in comparison to that obtained by VPR.

It is well known that the endothelium is an important regulator of the vascular tone, releasing endothelium-derived relaxing factors, including NO, COX-derived products, and endothelium-derived hyperpolarization factors (EDHFs) [13, 14]. To investigate the participation of these factors in the vasorelaxant effect induced by VPR, we performed experiments in the absence of functional endothelium. Under these conditions, VPR vasorelaxant maximum response was not significantly altered; however, the potency of VPR was reduced in absence of vascular endothelium. Therefore, to investigate the role of endothelium-derived relaxing factors, such as NO and COX-derived products, assays were performed with L-NAME (eNOS blocker) or indomethacin (COX inhibitor). In these conditions, vasorelaxant response mediated by VPR was significantly reduced, suggesting that VPR inhibit the releasing of endothelial NO and prostanoids. 

This suggests that the presence of a functional endothelium is important but not essential to the relaxation response mediated by VPR, and that an endothelium-independent pathway is also involved in this effect, which led to the subsequent experiments exploring VPR-mediated vasorelaxation in the absence of the endothelium.

Vascular tone, the contractile activity of vascular smooth muscle cells (VSMCs), is the major determinant of blood flow resistance through the circulation. Therefore, vascular tone plays an important role in the regulation of blood pressure and the blood distribution [15]. Due to the fact that many drugs exert their antihypertensive effects by decreasing peripheral vascular resistance, by direct action on vascular smooth muscle, we investigated the mechanisms underlying endothelium-independent relaxation induced by VPR in mesenteric artery rings.

The literature reports that VSMC contraction induced by high extracellular K^+^ concentration is mediated by an increase in membrane depolarization and, consequently, an increase in Ca^2+^ influx through voltage-dependent calcium channel (Ca_*v*_) [16]. Furthermore, the sustained contraction generated by a high external K^+^ concentration may also be mediated by Ca^2+^ release from the sarcoplasmic reticulum [17], resulting in Ca^2+^ entry through store-operated channels (SOC) and/or transient receptor potential (TRP) channels [18].

Endothelium removed mesenteric rings were precontracted with a depolarizing K^+^ solution (KCl 80 mM) in order to observe VPR responses before nonspecific generated contractions and averting activation of membrane receptors. In the presence of this depolarizing solution, the concentration-response curve induced by VPR was significantly shifted to the right, when compared to the response of VPR in the presence of Phe, changing both the efficacy and potency of VPR.

Based on these preliminary results, we speculate that this VPR-mediated vasorelaxant effect requires activation of potassium channels (K^+^ channels) in VSMCs. The opening of these channels in VSMCs results in an increase in K^+^ permeability, leading to K^+^ efflux. The exit of K^+^ from the VSMC induces membrane hyperpolarization and consequently the closing of voltage-operated Ca^2+^ channels, leading to vasorelaxation [19, 20]. Membrane potential regulation in VSMCs is an important factor in the maintenance of vascular tone. Additionally, K^+^ channels are effector proteins that contribute to membrane potential regulation in electrically excitable cells, such as VSMCs. Therefore, drugs that activate these channels could be beneficial in hypertension treatment [21].

Thus, to evaluate the involvement of K^+^ channels in VPR-mediated vasorelaxant response, experiments were performed in endothelium-denuded arterial rings incubated with KCl 20 mM. This procedure partially decreases membrane efflux of K^+^ due to the increase in extracellular K^+^ concentration from 4 mM to 20 mM, resulting in a reduction in the electrochemical gradient [22]. Under these conditions, the maximum response to VPR was significantly attenuated, which was also observed when rings were exposed to KCl 80 mM, suggesting the participation of K^+^ channels in vasorelaxation induced by VPR.

To confirm the involvement of K^+^ channels, the preparations were pretreated with TEA (3 mM), a nonselective K^+^ channel blocker [23]. In the presence of this pharmacological tool, the vasorelaxant response induced by increasing concentrations of VPR was significantly attenuated, corroborating our previous observation that VPR appears to have a significant effect on the open probability of K^+^ channels in mesenteric artery rings.

Several types of K^+^ channels are expressed in membrane of VSMCs, and each of these channels plays an important role in the control and maintenance of the contractile tone of the arterial muscle [24]. In VSMCs, the variety of K^+^ channels subtypes identified includes voltage-dependent K^+^ (K_*v*_) channels, whose activity is increased upon membrane depolarization and are important regulators of vascular smooth muscle membrane potential in response to a depolarizing stimulus [25]; large-conductance Ca^2+^-activated K^+^ (BK_Ca_) channels, which respond to changes in the concentration of intracellular Ca^2+^, regulating membrane potential, and have an important role in control of myogenic tone in smaller resistance arteries [26]; ATP-sensitive K^+^ (K_ATP_) channels, which respond actively to changes in cellular metabolism and are targets of a variety of relaxing stimuli [27]; and inward rectifier K^+^ (K_IR_) channels, regulating membrane potential of various types of resistance vessels of small diameter [28].

Thus, to investigate the possible participation of these different K^+^ channels subtypes, experimental preparations were separately preincubated with different selective blockers of these channels. In the presence of a K_ATP_ channels blocker, Glib (10 *μ*M), or a K_*v*_ channels blocker, 4-AP (1 mM), the maximum effect of VPR was not changed. The same response occurred in rings preincubated with BaCl_2_ (30 *μ*M), K_ir_ channels blocker, suggesting that K_ATP_, K_*v*_, and K_ir_ channels do not participate in VPR-mediated vasorelaxation.

The K_Ca_ channels are divided into a subfamily of small (SK_Ca_), intermediate (IK_Ca_), and large (BK_Ca_) conductance. The BK_Ca_ channels are expressed in vascular tissues with a high density; they are a major component that mediates the degree of depolarization and contraction in vascular smooth muscle; they are preferentially expressed in vascular smooth muscle, while the SK_Ca_ and IK_Ca_ are expressed in a variety of cell types such as secretory epithelial cells, fibroblasts, T lymphocytes, melanoma cells, granulocytes, macrophages, erythrocytes, cultured cell lines, and endothelial cells [29, 30]. BK_Ca_ channels act by inhibiting the increase of intracellular Ca^2+^. Furthermore, several recent findings support the importance of BK_Ca_ channels in the regulation of vascular smooth muscle tone and regulation of blood pressure [31].

The data shows that VPR-mediated vasorelaxation at the highest concentration was significantly attenuated in rings preincubated with TEA (1 mM), which preferentially blocks BK_Ca_ channels at concentrations lower than 1 mM [32]. These results suggest that BK_Ca_ channels mediate VPR-induced vasorelaxation, demonstrating that in addition to the neuronal actions, VPR exhibits important peripheral vascular effects as well.

Therefore, *in vivo* experiments were performed to evaluate VPR effects on cardiovascular parameters in nonanesthetized normotensive rats, in order to avoid the influences of anesthesia and postsurgical stress. In these animals, VPR induced a hypotensive response associated with tachycardia. The hypotensive effect caused by VPR may be due to decreased peripheral vascular resistance, and the tachycardia may be a reflection of the decrease in blood pressure.

Taken together, these results demonstrate that VPR induce vasodilatation probably involving K^+^ channel activation, most likely through BK_Ca_ channels, in smooth muscle inner from rat superior mesenteric artery. Furthermore, endothelium-derived relaxing factors (NO and prostanoids) are involved in endothelium relaxation dependent on VPR.

## Figures and Tables

**Table 1:** Participation of K^+^ channels in the vasorelaxation induced by VPR in mesenteric artery rings precontracted with phenylephrine.

Condition experimental	Maximum response (MR) (% relaxation)	EC_50_ (95% confidence interval)	*n*
Intact endothelium	75.43 ± 4.1	5.97 (3.8–9.3)	6
Denuded endothelium	70.33 ± 4.6^#^	39.6 (27.2–57.6)*	5
KCl (80 mM)	40.00 ± 8.2^##^	92.9 (62.1–138.9)**	5
KCl (20 mM)	50.48 ± 6.0^##^	21.2 (11.4–39.2)^ns^	5
TEA (3 mM)	46.79 ± 6.1^##^	53.9 (35.7–81.3)^ns^	8
TEA (1 mM)	49.33 ± 6.4^##^	29.9 (16.0–56.0)^ns^	7
4-AP (1 mM)	64.88 ± 5.1^ns^	24.8 (15.4–40.2)^ns^	5
Glib (10 *μ*M)	64.32 ± 3.5^ns^	10.7 (6.6–17.3)^ns^	5
BaCl_2 _(30 *μ*M)	63.14 ± 6.1^ns^	12.7 (7.2–22.3)^ns^	5

Values are expressed as mean ± SEM; unpaired Student's *t*-tests were used to examine the difference between denuded endothelium and intact endothelium; one-way ANOVAs with the addition of Bonferroni's multiple comparisons post hoc test were used to compare denuded endothelium (control) with different inhibitors groups. ^#^No significance versus intact endothelium, *P < 0.05 versus intact endothelium; **P < 0.01 versus denuded endothelium; ^##^ *P* < 0.01 versus denuded endothelium; ^ns^no significance versus denuded endothelium.

## Abbreviations

4-AP: 4-Aminopyridine
Ach: Acetylcholine
cAMP: 3.5-Cyclic adenosine monophosphate
BK_Ca_: Large-conductance calcium-activated K^+^ channel
BaCl_2_: Barium chloride
Ca_*v*_: Voltage-dependent calcium channel
COX: Cyclooxygenase
DMSO: Dimethyl sulphoxide
EDHF: Endothelium-derived hyperpolarization factor
Glib: Glibenclamide
HR: heart rate
IK_Ca_: Intermediate-conductance calcium-activated K^+^ channel
K_ATP_: ATP-sensitive K^+^ channel
K_ir_: Inward rectifier K^+^ channel
K_*v*_: Voltage-dependent K^+^ channel
MAP: Mean arterial pressure
MR: Maximum response
NO: Nitric oxide
PAF: Platelet-activating factor
Phe: Phenylephrine
K^+^ channel: Potassium channel
SEM: Standard error of mean
SK_Ca_: Small-conductance calcium-activated K^+^ channel
SOC: Store operated channels
TEA: Tetraethylammonium
TRP: Transient receptor potential
VPR: Roots from the *Valeriana prionophylla *
VPF: Leaves from the *Valeriana prionophylla *
VSMC: Vascular smooth muscle cells.
